# Supplementary material for: Indole-3-Carbinol Inhibits Citrobacter rodentium Infection through Multiple Pathways Including Reduction of Bacterial Adhesion and Enhancement of Cytotoxic T Cell Activity
Source: Nutrients. 2020 Mar 27;12(4):917. doi: 10.3390/nu12040917 (PMC7230886; doi:10.3390/nu12040917)
Supplement: Supplementary file 1 [file nutrients-12-00917-s001.pdf]

Supplementary Materials

Indole-3-Carbinol Inhibits *Citrobacter Rodentium* Infection through Multiple Pathways Including Reduction of Bacterial Adhesion and Enhancement of Cytotoxic T Cell Activity

Yanbei Wu[1,2,3,4], Qiang He[4], Liangli Yu[5], Quynhchi Pham[3], Lumei Cheung[3], Young S. Kim[6], Thomas T.Y. Wang[3*] and Allen D. Smith[3*]

[1] China-Canada Joint Lab of Food Nutrition and Health (Beijing)， Beijing Technology &Business University, Beijing 100048, P. R. China;

[2] Beijing Advanced Innovation Center for Food Nutrition and Human Health, Beijing Technology &Business University, Beijing 100048, P. R. China;

[3] Diet, Genomics, and Immunology Laboratory, Beltsville Human Nutrition Research Center, USDA-ARS, Beltsville, MD 20705, USA

[4] College of Biomass Science and Engineering, Sichuan University, Chengdu 610065, P. R. China

[5] Department of Nutrition and Food Science, University of Maryland, College Park, MD 20742, USA

[6] Nutritional Science Research Group, Division of Cancer Prevention, National Cancer Institute, National Institutes of Health, Bethesda, MD 20892, USA

*Corresponding author: Tom.wang@ars.usda.gov (Thomas T.Y. Wang); Allen.Smith@ars.usda.gov (Allen D. Smith); Tel: (301) 504-8459 (Thomas T.Y. Wang); (301) 504-8577 (Allen D. Smith)

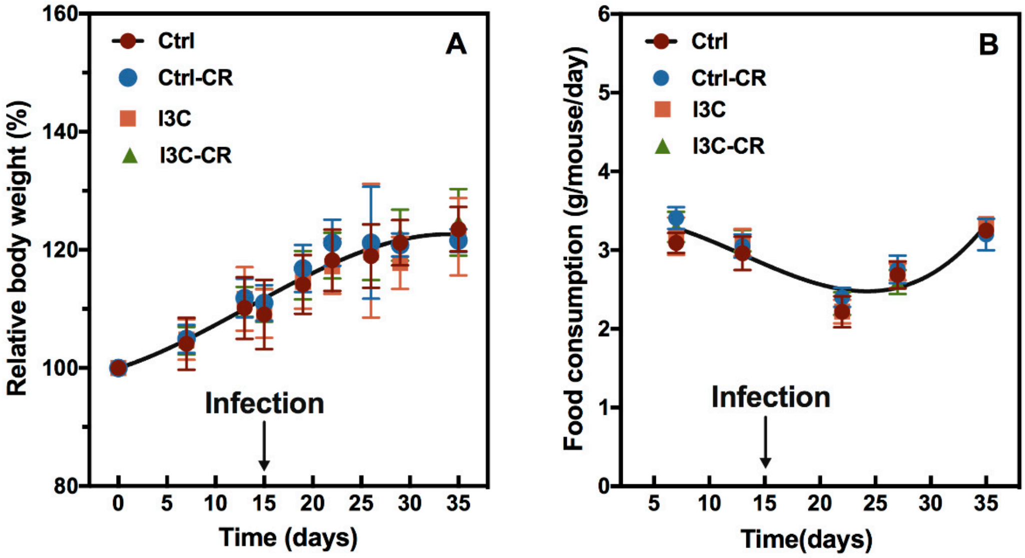

Figure S1. Effect of *Cr* infection on body weight (A) and food consumption (B) in uninfected or infected mice fed control or I3C diet. The body weight of mice on different days was normalized to each animal's body weight on day 0. Infection with *Cr* was initiated on day 15 after start of the diet. Results were expressed as mean +/- SD (n=8).

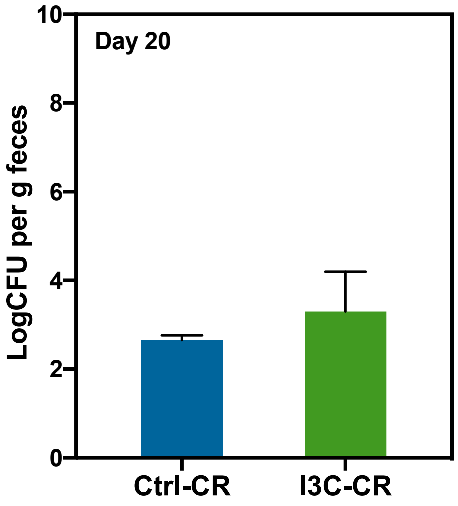

Figure S2. Fecal excretion of *Cr*. The feces of mice were collected on day 20 after infection and the Log cfu/g feces determined set. Results were expressed as mean +/- SD (n=8).

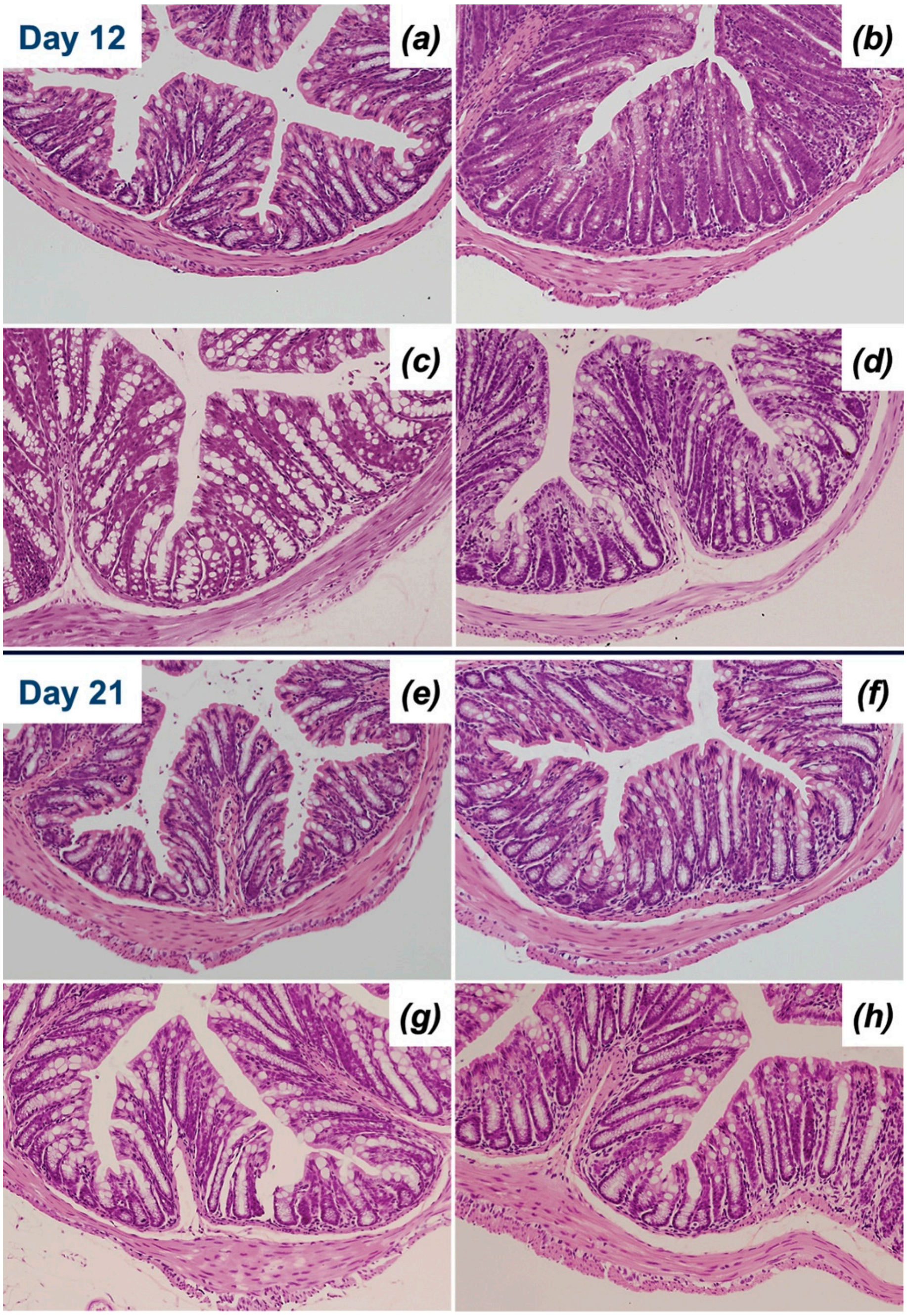

Figure S3. Representative images of H&E stained colon sections of uninfected and *Cr*-infected mice fed control or I3C diet. The colon tissues were collected from day 12 (a, b, c, d) and day 21(e, f, g, h) after infection. Original magnification ×100. (a, e): uninfected mice fed with control diet (Ctrl); (b, f): infected mice fed with control diet (Ctrl-*Cr*); (c, g): uninfected mice fed with I3C diet (I3C); (d, h): infected mice fed with I3C diet (I3C-*Cr*). The colonic hyperplasia and the goblet cell loss were found in the mice infected with *Cr*, the damage of colon recovered on 21 days 'post-infection. I3C could ameliorated the damage caused by the infection on day 12.
